# Xenotransplantation and the Role of the Patient Voice

**DOI:** 10.34067/KID.0000000000000310

**Published:** 2023-11-16

**Authors:** Kevin John Fowler, Vanessa A. Evans, Vineeta Kumar, Jeff Ross

**Affiliations:** 1 The Voice of the Patient, Inc.; 2 Fresenius Medical Care; 3 UAB SBS: The University of Alabama at Birmingham College of Arts and Sciences; 4 MiroMatrix

**Keywords:** kidney, kidney disease, kidney failure, kidney transplantation, patient-centered care, quality of life, renal dialysis, renal progression, USRDS (United States Renal Data System)

On behalf of the patient community, we are very grateful for the advances in xenotransplantation. The demand for kidney transplantation far exceeds the supply of available organs. Although increasing living donation and reducing kidney discard rates are important steps, neither will solve the problem. Advances in xenotransplantation represent a significant investment in innovation to solve a global problem. Historically, investment in kidney transplantation innovation pales in comparison with other specialty areas such as oncology. The US Food and Drug Administration (FDA) approved seven oncology medications in 2022.^1^ By contrast, belatacept was the last FDA-approved primary treatment of kidney transplant rejection in 2011.^2,3^ We hope that the excitement surrounding xenotransplantation results in renewed interest in medical school graduates and scientists pursuing transplantation as a career. There has been a marked decline in graduates pursuing transplantation. Xenotransplantation may be the spark that reignites interest given the opportunity to be on the leading edge of scientific innovation.^4,5^

Xenotransplantation has provided hope to the patient community. It is in preclinical development, and clinical trials have yet to begin. We participated in a Kidney Health Initiative project “*Understanding What Patients and Families Living with Kidney Failure Want and Need to Know about Kidney Transplant Research Possibilities*.” This included a series of virtual interviews conducted by the American Institutes for Research with 20 people living with kidney failure and ten care partners recruited from a diverse panel of patients. The purpose was to understand what people with kidney failure and their families want and need to know about kidney transplant innovations, such as xenotransplantation.

The project team developed comprehensive interview guides with questions in four topic areas: personal experience with kidney failure treatments, awareness of xenotransplantation, perceptions about xenotransplantation, and perceptions about future first-in-human xenotransplantation studies.

A summary of key findings are provided below.

## Perceptions of Xenotransplantation

- Overall reactions: Participants had a mix of opinions and reactions when first introduced to the concept of xenotransplantation; 25 participants (83%; 17 patients and eight care partners) expressed positive reactions to xenotransplantation. Participants commented that xenotransplantation could provide another solution for people who need a kidney transplant. Approximately 17% of participants (three patients and two care partners) had strong negative reactions to the concept.

One patient quote captures the potential promise of xenotransplantation:

“The supply of human kidneys is very low and the demand high. If that supply could be augmented with xeno(transplant) kidneys, then it would save a lot of lives.”—Patient (peritoneal dialysis)

- Concerns: Participants' concerns included skepticism or uncertainty over whether the procedure worked; questions about how pigs were being sourced, prepared, and treated; and the effect of a xenotransplant on their other health problems. Many participants expressed concerns over not wanting to be the initial test subject or “guinea pig.”

The potential to solve a global problem requires a recognition that much remains unknown about xenotransplantation:

The concern would be that it’s a new procedure and there’s…nobody with a successful transplant that way…Until I heard a couple of people had this successful transplant that way, I’d be a little apprehensive.”—Patient (in-center hemodialysis)

- Information needs: More than half of participants wanted additional information about xenotransplant research, including length of the research to date, results and statistics from previous studies, transparency on the progress of research to date, explanations of how pig kidneys are being modified for this procedure, and when the xenotransplant procedure will be widely available.

## Perceptions of Clinical Trials of Xenotransplantation

- General perceptions and experience of clinical trials: Participants were generally supportive of clinical trials, but expressed barriers to their own participation in trials. Thirteen participants (seven patients and six care partners) had participated in a clinical trial.
- Perceptions of xenotransplantation clinical trials: Most patients and care partners reported positive reactions to potential future clinical trials for xenotransplantation and noted the importance of clinical trials to advance medicine.
- Likelihood of participating in a xenotransplantation clinical trial, if offered: Fourteen patients and care partners (47%) were neutral to volunteering for a clinical trial of xenotransplantation, if available. Nine patients and care partners (30%) were likely or extremely likely to volunteer because this would be a better option to receive a kidney than continuing to wait for a kidney from a living donor. Seven participants (23%) were unlikely or extremely unlikely to participate in a clinical trial for xenotransplantation.

### A Call to Action to Advance Xenotransplantation Clinical Development

These patient and care partner insights represent a snapshot of the community with kidney failure and highlight a call to action to advance xenotransplantation further to clinical development.

We recommend that clinical development efforts that advance xenotransplantation include:

1. Patient voice

Patient voice must be on parity with all stakeholders in clinical development. The FDA guidance on Patient Focused Drug Development states that the patient community must be engaged as early as possible and on a consistent basis. Xenotransplant companies should be directly engaging with patients and their care partners in the preclinical and clinical development stages.

The Standardized Outcomes in Nephrology (SONG) Initiative demonstrated the discordance between patient and physician preferences.^6^ The Xenotransplantation workshop hosted by the National Kidney Foundation in 2021 highlighted the many unknown risks in xenotransplantation. This underscores the importance of implementing the patient focused drug development approach to facilitate patient preferences and manage the unknown.

The SONG Initiative created a collaborative environment where the patient voice is heard and acted upon. A SONG Initiative focused on xenotransplantation could ensure that patient preferences are prioritized, reducing the risk of physician and innovator discordance about what patients and their families need to know about xenotransplantation.

1. Patient engagement strategy

Over ½ of the interviewed participants wanted additional information about xenotransplantation. People with kidney failure do not have the highest levels of patient activation.^7^ When designing a patient engagement strategy for the trials, consideration must be given on how to communicate xenotransplantation concepts so that patients and their families can make a fully informed decision while acknowledging gaps in scientific understanding.

Patients must be informed of potentially competing trials. For example, the wearable artificial kidney and implantable artificial kidney are two emerging technologies that may offer improvement in quality of life for patients on dialysis.^8^ Patients with kidney failure deserve the right to be educated on all available clinical trials to ensure they have made a fully informed decision.
